# Regularity is not a key factor for encoding repetition in rapid image streams

**DOI:** 10.1038/s41598-019-39697-y

**Published:** 2019-05-03

**Authors:** Evelina Thunell, Simon J. Thorpe

**Affiliations:** Centre de Recherche Cerveau et Cognition (CerCo), Centre National de la Recherche Scientifique (CNRS), Université Paul Sabatier, Toulouse, France

**Keywords:** Cognitive neuroscience, Learning and memory, Visual system

## Abstract

Human observers readily detect targets and repetitions in streams of rapidly presented visual stimuli. It seems intuitive that regularly spaced repeating items should be easier to detect than irregularly spaced ones, since regularity adds predictability and in addition has ecological relevance. Here, we show that this is not necessarily the case, and we point out the intrinsic difficulty in addressing this question. We presented long RSVP streams of never-before-seen natural images containing repetition sequences; an image appearing six times interleaved by one or more non-repeating distractors, and asked participants to detect the repetitions and to afterwards identify the repeated images. We found that the ability to detect and memorize repeated images was preserved even with irregular sequences, and conclude that temporal regularity is not a key factor for detection and memory for repeating images in RSVP streams. These findings have implications for models of repetition processing.

## Introduction

When studying for an exam or trying to learn a new word, repetition certainly helps. In certain cases, mere repetition without task relevance or explicit instructions can even be enough for plasticity and learning. This is the case in “statistical learning”, where regularities in the sensory input are stored implicitly without conscious awareness^1^. Both in the auditory^2^ and in the visual^3^ domains, human observers discriminating between looped (two auditory noise snippets or visual noise sequences presented back-to-back) and uncorrelated stimuli show preferential processing of looped stimuli that re-occur in several trials. This typically happens unknowingly to the participant, again pointing to some automatic or unconscious component of the underlying mechanisms. To further explore the efficacy of repetition processing on short time-scales, we recently developed a novel paradigm based on rapid serial visual presentation (RSVP)^4,5^ aimed at testing the capacity for detecting and remembering repeating natural images embedded in continuous streams of never-before-seen items. RSVP is ubiquitous in the literature on visual processing. Since its appearance almost 50 years ago, RSVP has been used to demonstrate a wide range of effects including the attentional blink^6–10^, repetition blindness^11,12^, and more recently negative priming^13^ (but see also for example^14^) and enhanced memory for images that deviate in terms of emotional content from the rest of the stream^15^. Common to most of these paradigms is that participants are asked to report one or more pre-defined targets such as for example faces, or are required to remember all presented items. The streams typically comprise rather few items. In our new paradigm, the task is instead to spot repetitions in streams of hundreds or even thousands of images. The repeated images are chosen at random without any specific criteria and appear at random time points, meaning that it is not possible to foresee which image will become the next target. We previously showed that human observers can spot and remember repeating images under a wide range of conditions (Thunell, E. & Thorpe, S. J. (2019). Memory for repeated images in rapid-serial-visual-presentation streams of thousands of images. Psychological Science. Advance online publication. 10.1177/0956797619842251). Both detection and memory for repeated items was above chance already with two or three presentations of the repeated image and increased with the number of presentations up to a ceiling level at around 7. The image presentation rate also strongly influenced performance, with the slowest streams being the easiest and the difficulty increasing with the presentation rate, at least for low rates. At around 15 Hz, performance stabilized and remained above chance up to 120 Hz. This remarkable capacity has implications for computational models of repetition learning. Importantly, in our previous experiments the repetitions were always regularly spaced; each repeated image (target; T) was interspaced by either one or two non-repeated distractors (D), i.e. the sequences were of the form T-D-T-D-T-D-T or T-D-D-T-D-D-T-D-D-T. Here, we ask whether this regularity is a key factor for repetition perception. Are regularly and irregularly spaced repetition sequences equally easy to detect and remember, as predicted by for example spike-timing dependent plasticity (STDP; see explanation below) based models of learning^16^? Or does irregular spacing perturb the processing? The latter seems intuitive considering our instinctive liking of rhythmic patterns and their importance in natural sensory input –arising from for example animal and human locomotion, and the added predictability of regular stimulation. A deleterious effect of irregularity was also suggested by Rajendran, Harper, Abdel-Latif, & Schnupp^17^ in the auditory domain, and taken as evidence against STDP-like mechanisms. STDP is a Hebbian learning rule where synaptic strength varies as a function of the relative timing of pre- and post-synaptic spikes, capable of causing neurons to become sensitive to repeated input spike patterns^16,18–20^. It has recently been proposed as a possible mediator of statistical learning^21^, and could potentially support also the repetition perception in our paradigm.

The role of regularity in repetition detection is not trivial to address: It is important to note that when manipulating the regularity of a repetition sequence, other crucial aspects of the stimulus necessarily also change. For example, if we choose to preserve the length of the repetition sequence and introduce irregularity by jittering the target presentations within the sequence, new inter-target intervals are introduced which do not exist in the corresponding regular sequence. As we showed in our previous study, the length of these intervals can greatly influence performance (Thunell, E. & Thorpe, S. J. (2019). Memory for repeated images in rapid-serial-visual-presentation streams of thousands of images. Psychological Science. Advance online publication. 10.1177/0956797619842251). On the other hand, if we balance the total distribution of inter-target intervals, the sequence lengths will instead vary between regular and irregular sequences – a factor that might also in itself influence performance. Here, in an attempt to make the most complete comparison possible, we used both methods described above. Our results show that even with strongly irregular repetition sequences, the ability to detect and memorize repeated images is preserved.

## Results

**Figure 1:**
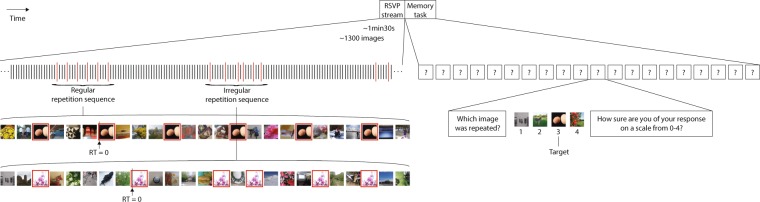
Stimuli. Each 15 Hz RSVP stream (left) contained over 1300 images. Here, a part of the stream is shown; each vertical line depicts an image. Each image was presented for 58.3 ms followed by an 8.3 ms blank. At random time points, an image was presented six times (longer red lines). In the Regular repetition sequence shown in detail, there are 3 distractors between each presentation of the target (the eggs). Note that there were also Regular repetition sequences with 1, 2, 4, and 5 distractors. In the Irregular repetition sequence, there is a varying number of distractors (1–5) between each presentation of the target (the pink magnolia flowers). In the Memory task that followed each RSVP stream (right), the repeated images had to be identified among sets of three non-repeated images from the same stream, along with a confidence rating. The red frames marking the targets were not present in the actual stimulation.

We recently developed a novel RSVP paradigm that we used to probe repetition perception as a function of the number of repetitions of an image, the number of distractors between repetitions, and the image presentation rate. Here, we adapted this paradigm to explore the effect of temporal regularity of the repetition sequences. We presented 15 Hz RSVP streams of never-before-seen natural images, each lasting about 1.5 minutes and containing 20 repetition sequences. A repetition sequence consisted of an image presented six times either in a regular fashion (with the same number of distractors between each presentation) or irregularly (with a varying number of distractors; Fig. 1). The task was to detect the repetitions (push a button every time a repetition sequence was spotted) and to afterward identify all 20 repetition targets in a 4-alternative forced choice (4AFC) task. The targets were chosen at random and independently of image content, meaning that there were no target-defining features. Further, the repetition sequences appeared at random time intervals (~3 s after the last one finished), making it impossible to foresee which image would become the next target.

### Repetition detection task

**Figure 2:**
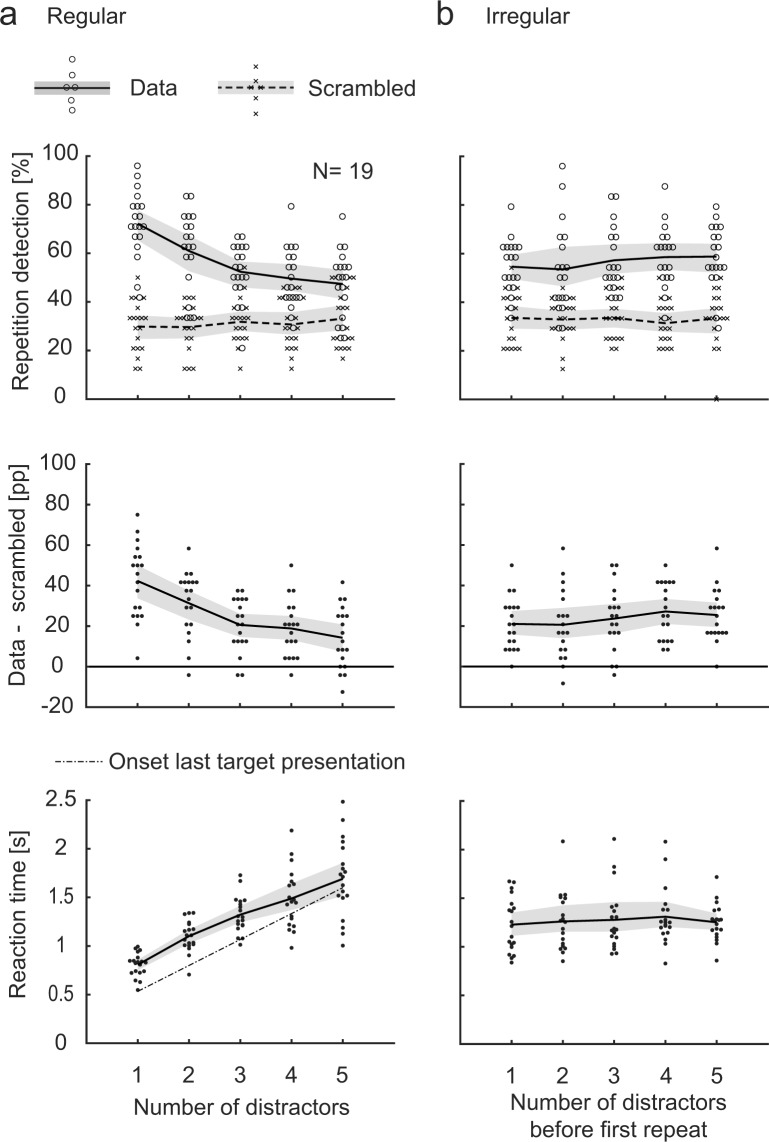
Repetition detection compared to scrambled data (chance level) and RTs. In the upper panel, the repetition detection performance is shown together with the corresponding results for the scrambled data. In the Irregular conditions, the data are somewhat arbitrarily plotted as a function of the number of distractors before the first repetition. The percentage point (pp) difference between the real and scrambled data shown in the middle panel can be taken as a chance-level corrected measure of the detection performance. In the lower panel, RT = 0 is the onset of the second presentation (the first repetition) of the target. There were 13.4 ± 1.0 button-presses per block (mean ± SD across participants); 2.2 ± 2.1% of all trials contained more than one response in the response interval. Each data point depicts the mean per participant and condition. The lines indicate the mean across participants, and the shaded areas are 95% bootstrap confidence intervals.

First, we note that our participants were able to detect both Regular and Irregular repetition sequences: The detection rate was well above the “chance level” computed by assigning random time stamps to the button-presses in all conditions (Fig. 2; the 95% confidence intervals (CIs) for the difference between the real and the scrambled data do not span zero). Note that the Irregular “conditions” are here created somewhat arbitrarily based on the number of distractors in the *first* inter-target interval of each trial, in order to make a comparable plot as for the Regular conditions. The task seems to have been easiest with only 1 distractor and increasingly difficult with more distractors, as confirmed by a one-way ANOVA showing a significant effect of number of distractors (F(4, 90) = 10.5, p = 5.4 × 10^−7^; post-hoc Tukey-Kramer testing revealed significant differences between the 1 distractor condition and the 3, 4, and 5 distractor conditions (p values = 2.4 × 10^−4^, 2.0 × 10^−5^, and 2.5 × 10^−6^, respectively), and between the 2 and 5 distractor conditions (p = 0.024)). This effect is not due to a speed-accuracy trade-off since the corresponding RTs increased with the number of distractors (Fig. 2a, lower panel).

**Figure 3:**
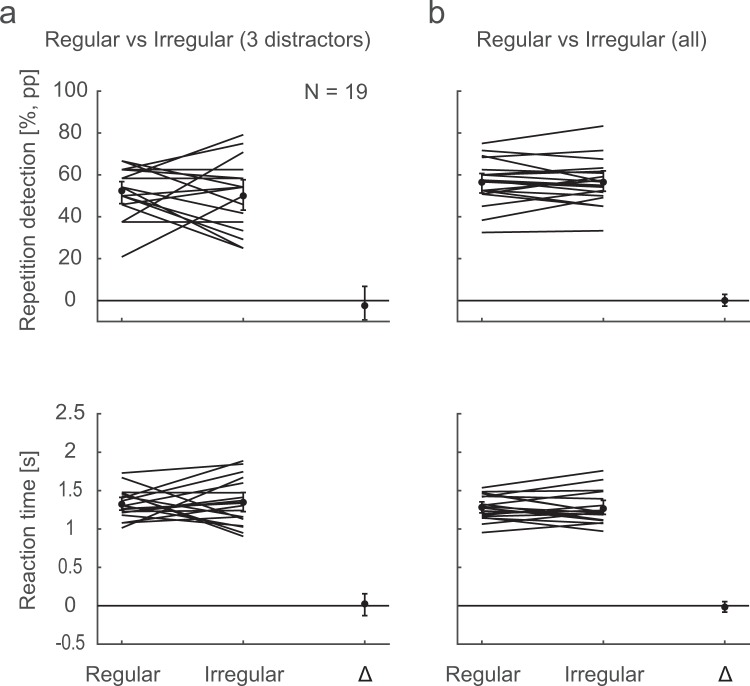
Repetition detection for Regular vs. Irregular sequences. In (**a**), repetition detection (upper panel) and RTs (lower panel) are shown for the Regular 3-distractor condition and an equal-size random sub-sample of the Irregular trials, together with the difference Irregular-Regular. Each line depicts the mean per participant and condition, and the dots mark the overall means. In (**b**), all Regular are contrasted against all Irregular trials. The error bars are 95% confidence intervals.

As a first comparison between the performance for Regular and Irregular repetition sequences, we contrasted the Regular 3-distractor condition against a random sub-sample of equally many trials of the Irregular conditions, and found no apparent differences between the two groups: The CIs of the difference between the Regular and Irregular conditions span zero both for detection performance and RTs (Fig. 3a). The 95% CIs provide a range of plausible values of the true difference, as we would expect the CI to include the true value 95% of the time when replicating the experiment. Additional Bayesian analyses revealed moderate evidence for the null hypothesis (detection performance BF10 = 0.279, RT BF10 = 0.248; see Supplementary material for details for all Bayesian tests). All repetition sequences included in this analysis have the same length, and the average number of distractors between repetitions is the same in the two groups. Any manipulation of the regularity of the repetition sequence will entail a possibly confounding modification of low-level properties, which is why we made also a second comparison, contrasting all Regular against all Irregular conditions. In this case, the *average* sequence length is the same in the two groups, and all inter-repetition intervals (1, 2, 3, 4, and 5 distractors) occur equally often in both. Again, the CIs of the difference span zero for both detection performance and RT (Fig. 3b). The CIs are here much smaller than for the first comparison, as there are about five times as many trials in this analysis. Bayesian analyses again showed moderate evidence for the null hypothesis (detection performance BF10 = 0.238, RT BF10 = 0.243).

### Memory task

**Figure 4:**
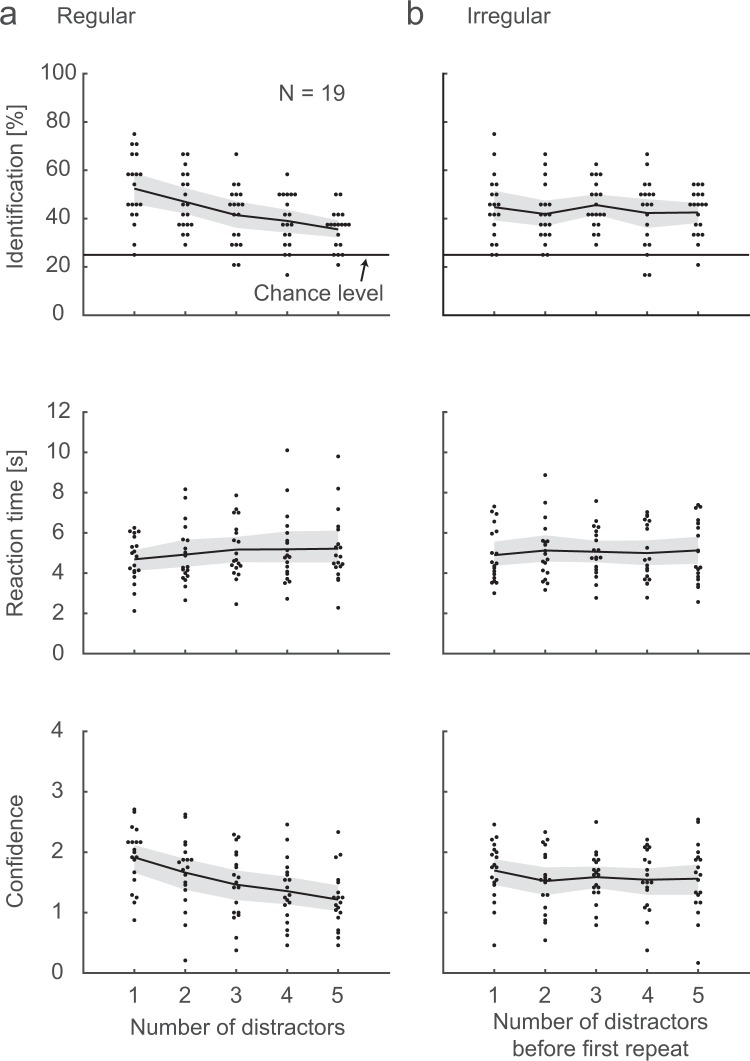
Memory task. In (**a**), identification performance (upper panel), RTs (middle panel), and confidence ratings (lower panel) are shown separately for each of the five regularly spaced repetition sequences. The graphs indicate the mean across participants. In the Irregular conditions (**b**), the data are plotted as a function of the number of distractors before the first repetition. The shaded areas are 95% confidence intervals.

Each RSVP stream was followed by a 4AFC Memory task where the participants were asked to identify all the repeated images from the stream (Fig. 1, right). As in the repetition detection task, the participants performed well above chance in all conditions (Fig. 4, upper panel; the CIs do not span zero). The performance again dropped with number of distractors in the Regular sequences (one-way ANOVA: F(4, 90) = 6.2, p = 1.8 × 10^−4^; post-hoc Tukey-Kramer testing revealed significant differences between the 1 distractor condition and the 3, 4, and 5 distractor conditions (p values = 0.037, 5.7 × 10^−3^, and 2.2 × 10^−4^, respectively), and between the 2 and 5 distractor conditions (p = 0.027)). The RTs were rather stable, except perhaps for a slight increase with the number of distractors (Fig. 4a, middle panel), speaking against a speed-accuracy trade-off. The confidence ratings mimic the identification performance, indicating that the participants had an accurate perception of the relative difficulty levels in the different conditions (Fig. 4, lower panel). As with the Repetition detection data, the Irregular trials were arbitrarily grouped according to the number of distractors in the *first* inter-target interval.

**Figure 5:**
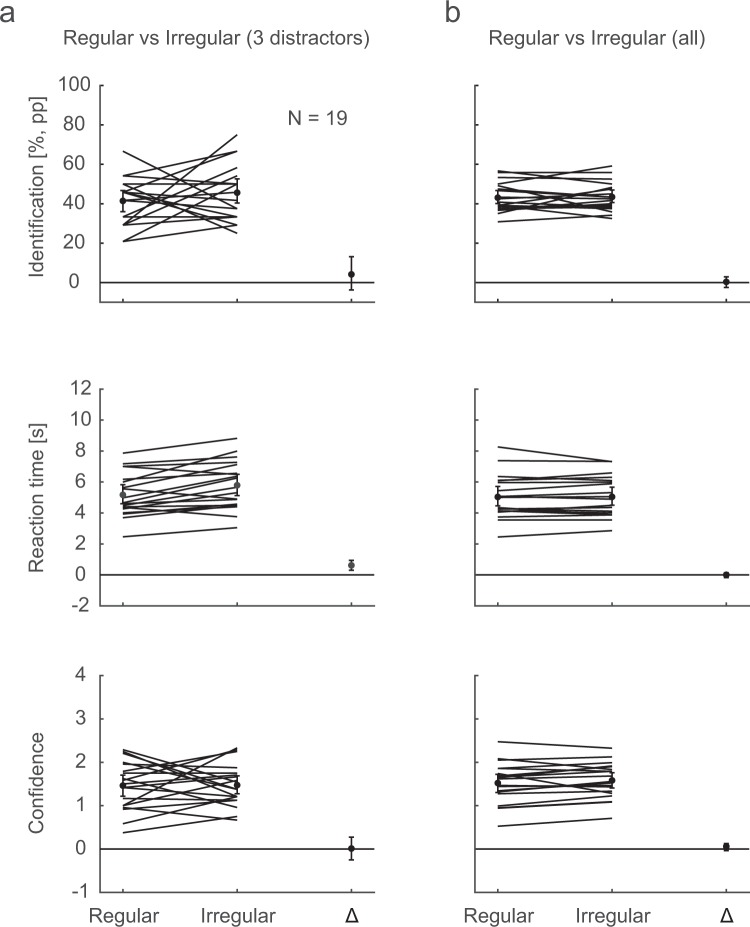
Memory for Regular vs. Irregular sequences. In (**a**), identification performance (upper panel), RTs (middle panel), and confidence ratings (lower panel) are shown for the Regular 3-distractor condition and an equal-size random sub-sample of the Irregular trials, together with the difference Irregular-Regular. Each line depicts the mean per participant and condition, and the dots mark the overall means. In (**b**), all Regular are contrasted against all Irregular trials. The error bars are 95% confidence intervals.

The first comparisons between Regular and Irregular trials (Regular 3-distractor condition against a random sub-sample of equally many trials of the Irregular conditions; Fig. 5) revealed comparable identification performance in the two groups, but faster RTs for Regular repetition sequences than for Irregular ones. Bayesian analyses showed anecdotal evidence for the null hypothesis for the identification performance (BF10 = 0.355) and strong evidence for a RT difference (BF10 = 20.8). In the second comparison (all Regular against all Irregular conditions), identification performance, RTs, and confidence ratings are similar in the two groups. The Bayesian analyses revealed moderate evidence for the null hypothesis for identification performance (BF10 = 0.244) and RTs (BF = 0.238), and anecdotal evidence for the null for the confidence ratings (BF10 = 0.561). Thus, the results largely resemble those for the Repetition detection task.

Last, we looked for signs of memory decay during the blocks. Since the order of target appearance was randomized in the Memory task, there are different delays between the appearances of the same target in the RSVP stream and the Memory task. Is there for example a recency effect (increased memory strength for the last targets in the stream) or a primacy effect (increased memory strength for the first targets in the stream)? We compared the identification performance for short versus long delays between the appearance of the same target in the RSVP stream and the Memory task (Supplementary Material, Fig. S1). The CIs of the difference span zero for performance, RTs and confidence ratings. Bayesian analyses of these differences revealed moderate evidence for a null effect for the identification performance (BF10 = 0.245) and confidence (BF10 = 0.250), and anecdotal evidence in the case of the RTs (BF10 = 0.340).

## Discussion

We recently revealed a remarkable capacity of the human visual system for detecting and remembering repeating images in RSVP streams of thousands of images (Thunell, E. & Thorpe, S. J. (2019). Memory for repeated images in rapid-serial-visual-presentation streams of thousands of images. Psychological Science. Advance online publication. 10.1177/0956797619842251). We showed that at 15 Hz, one single repetition can be enough for detection and memory, and that observers can do the task in fast streams at up to 120 Hz when each target is presented five times. Here, we modified the paradigm to include both regularly and irregularly spaced repetition sequences, in order to investigate whether temporal regularity is a crucial factor for repetition detection and memory. It is important to note that it is not trivial to isolate the effect of regularity, since it typically co-varies with other aspects of the stimulation. We made two different comparisons between regularly and irregularly spaced repetition sequences (equalizing first the sequence length, then the distribution of number of distractors between repetitions, between the two groups) and observed only minor behavioural differences with either method. Importantly, the stimulation contained equally many regularly as irregularly spaced repetition sequences, meaning that there was no advantage of preferentially processing either type. Our participants performed well above chance on both the repetition detection and the memory task in all regular and irregular conditions, indicating that the mechanisms supporting repetition processing in this paradigm do not strongly depend on regularity. In fact, behaviour for regular and irregular repetitions was comparable in both tasks except for faster identification RTs for the former in the 3-distractor comparison. We might add that even the authors, who were highly trained on regular repetition sequences, performed equally well with irregular as regular sequences in pilot studies (none of the authors participated in the experiment reported here). It should be noted that our Repetition detection task is conceptually similar to an n-back task, and even though classic n-back paradigms typically involve longer presentation times, both necessitate some form of short-term memory function for keeping the items stored for comparison.

In the Regular conditions, the difficulty in both tasks increased with the number of distractors between repeats (Figs 2a and 4a), in agreement with our previous finding of better performance for 1-distractor than 2-distractor streams. (Thunell, E. & Thorpe, S. J. (2019). Memory for repeated images in rapid-serial-visual-presentation streams of thousands of images. Psychological Science. Advance online publication. 10.1177/0956797619842251). We were surprised to find above-chance performance even when the repetitions were interspaced with as many as five distractors. Notably, in this condition the repetition detection involves a 6-back comparison and each target is masked by five other images (333 ms) before its next appearance.

In our previous study (Thunell, E. & Thorpe, S. J. (2019). Memory for repeated images in rapid-serial-visual-presentation streams of thousands of images. Psychological Science. Advance online publication. 10.1177/0956797619842251), any forgetting or lack of retention of the repeated images seemed to have happened to a similar extent for all the targets regardless of the number of other items intervening between presentation and prompting of a target, on the time-scale of minutes. As in that study, short and long delays are not balanced with respect to conditions in the current design, and so the results should not be over interpreted. Still, it is interesting to again notice the lack of apparent dependencies on trial order, a potential effect that merits further investigation. Previous RSVP studies research have revealed decaying target retention with the number of intervening test items and elapsed time on the time scale of seconds (but not from presentation order except possibly for the first and last picture)^4,22,23^.

Rajendran and colleagues^17^ asked a similar question about temporal regularity in the auditory domain. In their study, participants were presented with white noise sequences which either contained a repeated snippet (8 presentations), no repetition, or a transition to pink noise. The participants were instructed to push a button if they detected either a repetition or a transition. There were three irregular conditions (300 ms long repeated noise snippets separated by 200 ms ± 10, 50 or 100 ms; mean ± standard deviation), and four regular conditions (200, 300, 400 or 500 ms long repeated noise snippets appearing every 500 ms). The authors reported impaired performance with the most severe jitter (100 ms). The apparent discrepancy between our results and those of Rajendran and colleagues might be explained by several differences between the two studies. First, repetitions might be processed differently in the auditory as compared to the visual domain, or for noise stimuli as in the Rajendran study as compared to the meaningful stimuli used here. Further, in our study regular and irregular sequences were equally common, while in the study by Rajendran there were more regular than irregular trials. Although it is unlikely that this had any major influence, observers might implicitly learn the statistical properties of the stimulation and preferentially process regular sequences if they are more common. Rajendran and colleagues argue that STDP-like mechanisms alone cannot account for their results. The apparent lack of disruption of repetition processing by irregularity in our study, on the other hand, is in line with what would be predicted by STDP-based models of learning: With reasonably short repetition sequences, repetition processing with these algorithms does not depend on whether the items appear at regular or irregular intervals^16^.

Theories of periodic sampling of perception and attention propose rhythmicity at different frequencies between 4–10 Hz^24–26^. Based on these theories, we might expect certain particularly beneficial presentation rates of either the image stream or the targets in our paradigm. For example, visual attentional rhythms have been found at around 7 Hz^27–31^. Does this effect manifest as better performance around this frequency in our paradigm? In our previous study (Thunell, E. & Thorpe, S. J. (2019). Memory for repeated images in rapid-serial-visual-presentation streams of thousands of images. Psychological Science. Advance online publication. 10.1177/0956797619842251), where the image presentation rate varied between 4 and 120 Hz, there was no clearly emerging such “sweet spot”. In the current study, the presentation rate was fixed at 15 Hz and the repeated images appeared at 7.5, 5, 3.75, 3, and 2 Hz in the Regular conditions (for 1, 2, 3, 4, and 5 distractors, respectively). One could imagine a dependence not on the image presentation rate but on the rate of target appearance. However, the behavioural results seem to depend monotonically on this parameter across the tested values. In addition, the similar behavioural results for the regular and irregular sequences argues against any strong facilitating entraining specifically to temporally regular target occurrences. In line with this, Quek & Rossion^32^ found similar EEG responses to faces that appeared regularly as compared to irregularly in 12 Hz RSVP streams, and in addition no specific EEG correlate of missing faces in otherwise regular sequences.

To conclude, we found that the remarkable ability to process repetitions that we previously reported (Thunell, E. & Thorpe, S. J. (2019). Memory for repeated images in rapid-serial-visual-presentation streams of thousands of images. Psychological Science. Advance online publication. 10.1177/0956797619842251) is not restricted to temporally regular repetition sequences but generalizes also to irregular sequences. This finding adds to the constraints of plausible neural mechanisms supporting this capacity.

## Methods

### Participants

Twenty paid participants took part in the experiment, one of which was excluded due to overall poor performance. The remaining 19 (9 male) were aged 21–33 years (mean 27). All were naïve to the purpose to the study and had not previously seen any of the images or participated in any similar experiments. The procedures were in accordance with the Declaration of Helsinki and approved by the local ethics committee “Comité pour l’Evaluation de l’Ethique de l’INSERM” (CEEI protocol number 2015-004), and all participants gave written informed consent before starting the experiment.

### Stimuli and task

The paradigm used here is similar to that of (Thunell, E. & Thorpe, S. J. (2019). Memory for repeated images in rapid-serial-visual-presentation streams of thousands of images. Psychological Science. Advance online publication. 10.1177/0956797619842251), where we presented repeated items embedded in long Rapid Serial Visual Presentation (RSVP) streams and varied the number of presentations of the repeated images and the image presentation rate. Here, both these parameters were fixed, and we instead manipulated the regularity of the repetition sequences. We presented RSVP streams of natural images at 15 Hz; 58.3 ms image duration followed by an 8.3 ms gap (one screen refresh of background grey). The streams lasted between 86.9 and 91.2 s (1303–1368 images) with an average duration of 88.8 s (1332 images). In each of these streams of never-before-seen images, we embedded 20 repetition sequences; an image appearing six times interspaced with between one and five distractor images that were not repeated (Fig. 1). In half of the repetition sequences (Regular spacing conditions), the same number of distractors (1, 2, 3, 4 or 5) appeared between each of the six presentations of the repeated image. In the other half (Irregular spacing conditions), the number of distractors varied *within* each repetition sequence rather than across sequences. Here, the sequences each had 1, 2, 3, 4, *and* 5 distractors between the target presentations, and all 120 permutations of the order of these were used for each participant. It was not possible to anticipate when the next repetition sequence would occur, since there was an interval of variable length of between 2.5 and 3.5 s (37–52 images) between sequences and the images to be repeated were chosen at random. The participants were asked to push “s” on the keyboard every time they detected a repetition sequence, and to remember the repeated images for a subsequent Memory task. They were encouraged to push the button as soon as they noticed a repeating image, and they were reassured that this button-press could come after the repetition sequence, or even after the image stream had finished. We explicitly told the participants that all repetitions would appear in the form of relatively short repetition sequences, and that only one image at a time would be repeating. The Memory task that followed each RSVP stream was a four-alternative forced choice (4AFC) task where the repeated images had to be identified among sets of three other, non-repeated, images from the same stream. The participants were asked to rate their confidence for each response on a scale from 0 (guessing) to 4 (completely sure). They were informed that in each response frame, there was one image that had been repeated, and that the other three images had also appeared in the stream but only once. The order of the targets in the Memory task was randomized and did not match that of the RSVP stream. It was thus not directly useful for the Memory task to learn an ordered sequence of the targets in the RSVP stream. No feedback was given during the experiment.

To get an impression of the type of paradigm used here, the reader might try our freely available iPad game Brainspotting (https://itunes.apple.com/app/id1246763569), where the task is to identify regular repetitions in short RSVP sequences.

Each participant took part in one experimental session comprising 12 blocks (image stream with accompanying Memory task). The 120 permutations of the Irregular conditions and 24 instances of each of the five Regular conditions were presented in a random order and were thus not balanced across blocks. This design resulted in equally many Regular and Irregular spacing trials (120 of each). Before starting the experiment, the participants practiced on six blocks containing five or six repetitions sequences each at 2, 8 and finally 15 Hz. The experimenter was in the room during the practice and gave verbal feedback. The participants were informed that the image streams would last longer than during the practice. The whole session including practice lasted 1–1.5 h.

### Procedure and stimulus details

The images were a randomly chosen subset of the ImageNet database training set^33^ (http://image-net.org/), cropped symmetrically from all sides to square shape and equalized in resolution to 150 * 150 pixels. Each participant viewed approximately 16.000 different images. No images other than the repeated images and the images in the response frames were presented more than once to any participant. All images were equally likely to be used as a target, and which images were targets varied between participants.

The participants were seated in a dimly lit room (~0.3 cd/m^2^) approximately 75 cm from the screen. The images were 8.3 cm wide (~6.3 degrees of visual angle). The RSVP streams were presented in the middle of the screen. The four images in the response frames were centred on the horizontal meridian of the screen, separated by approximately one-third of the image width. The background grey was chosen to match the average grey-level of a sample of 14.000 randomly chosen images from the ImageNet subset (grey-level 114 out of 255). Both the RSVP streams and the Memory tasks were preceded by a reminder of the task, which disappeared when the participants launched the next step by pushing the space bar. Before each stream, the block number was indicated. The participants were instructed to look at the image streams, but were free to fixate any part of the images. They were encouraged to take short breaks between the blocks, when needed. Eye movements were not recorded.

### Apparatus

The stimuli were presented on a BenQ XL2411Z monitor (120 Hz refresh rate, resolution 1920 × 1080 pixels, 24”), controlled by a PC. The reliability of the screen refresh rate and the stimulus generating script were verified using a photodiode connected to an oscilloscope.

### Analysis

Since the RSVP stimulation was continuous and there were no response intervals indicated to the participants, and in addition the conditions were mixed, signal detection theory based analyses of the button-presses would not have been trivial. (See for example^34^ for a discussion of older methods and a new alternative for assessing detection sensitivity per block in fast or continuous stimulation). Instead, we considered a repetition sequence detected if there was one and only one button-press in an interval between 200 and 2500 ms after the first repetition (second presentation) of the target. The onset of the second presentation was defined as reaction time (RT) = 0. To get a meaningful assessment of the performance, the results were compared to a “chance-level” computed by performing the same analysis on scrambled data where, for each participant and block, the button-presses were given random time stamps.

We defined the delay between the appearances of a particular target in the RSVP stream and in the Memory task as the number of intervening targets in the stream and in the Memory task, plus one. The data were then split in half with respect to this delay and averaged within the two groups in order to contrast short and long delays. The very longest and shortest delays were not included since they were uncommon. Short delays were 3–20 and long were 21–37 trials. Note that the data is pooled over the conditions in a random, non-balanced, way in this analysis.

For analysis and stimulus generation we used Matlab and Psychtoolbox-3^35^. The 95% confidence intervals (CIs) of the participant means were computed using the Matlab bootci function with 2000 iterations and the default bias corrected and accelerated percentile method. For the Bayesian t-tests, we used the freely available software JASP^36^.

## Supplementary information

Fig. S1

## Data Availability

Neither of the experiments reported in this article was formally preregistered. The modified images from the ImageNet database training set are available at the open science framework https://osf.io/t7ej9/?view_only=ead6d92f5c8b4e90891edfc0c136b21c. Raw data and the details of the Bayesian analysis are available at https://osf.io/m4fy6/?view_only=730253996a6e489f82d393a3751ca1be.
