# Supplementary material for: Regularity is not a key factor for encoding repetition in rapid image streams
Source: Sci Rep. 2019 May 3;9:6872. doi: 10.1038/s41598-019-39697-y (PMC6499888; doi:10.1038/s41598-019-39697-y)
Supplement: Supplementary file 1 — Fig. S1 [file 41598_2019_39697_MOESM1_ESM.docx]

Supplementary material

Regularity is not a key factor for encoding repetition in rapid image streams

Evelina Thunell*, Simon J. Thorpe

Centre de Recherche Cerveau et Cognition (CerCo), Centre National de la Recherche Scientifique (CNRS), Université Paul Sabatier, Toulouse, France

*Corresponding author: ET, evelina.thunell@cnrs.fr

**
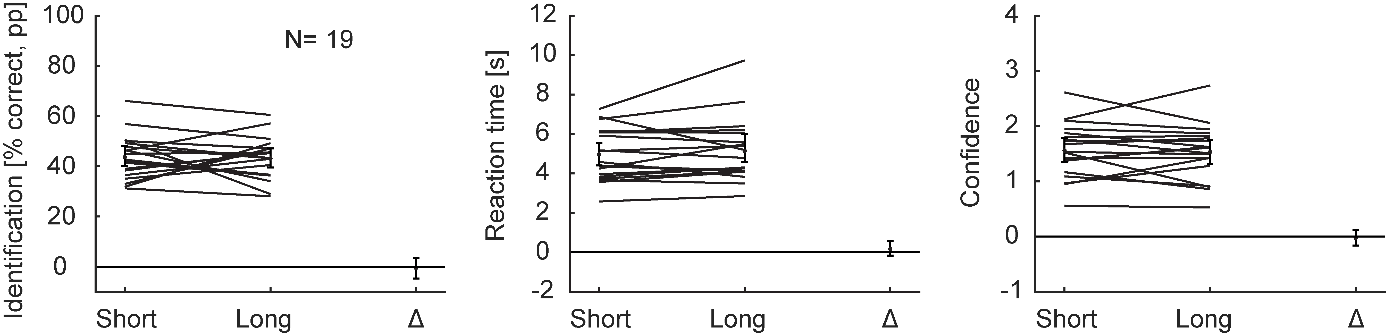
**

**Fig. S1.** Single-participant Memory task results for short vs. long trial delays. Identification performance (left), RTs (middle) and confidence ratings (right) are shown as a function of the delay between the appearance of the same target in the RSVP stream and the Memory task. Each line depicts a participant, and Δ symbolizes the difference long – short delays. All conditions are pooled (in a non-balanced way; see Methods). The error bars are 95% confidence intervals.
